# Supplementary material for: HIVprotI: an integrated web based platform for prediction and design of HIV proteins inhibitors
Source: J Cheminform. 2018 Mar 9;10:12. doi: 10.1186/s13321-018-0266-y (PMC5845081; doi:10.1186/s13321-018-0266-y)
Supplement: Supplementary file 2 — Additional file 2. Source code of HIVProtI web server. [file 13321_2018_266_MOESM2_ESM.zip › HIVprotI_Source-code/hivprotI/weka-3-6-6/doc/weka/clusterers/forOPTICSAndDBScan/OPTICS_GUI/SERFileFilter.html]

SERFileFilter


---


|  |  |  |
| --- | --- | --- |
| **Overview** \| **Package** \| **Class** \| **Tree** \| **Deprecated** \| **Index** \| **Help** \| **Weka's home** | |  |
| **PREV CLASS**   **NEXT CLASS** | **FRAMES**    **NO FRAMES**     **All Classes** |  |
| SUMMARY: NESTED \| FIELD \| CONSTR \| METHOD | DETAIL: FIELD \| CONSTR \| METHOD |  |


---


## weka.clusterers.forOPTICSAndDBScan.OPTICS\_GUI Class SERFileFilter

```
java.lang.Object
  javax.swing.filechooser.FileFilter
      weka.clusterers.forOPTICSAndDBScan.OPTICS_GUI.SERFileFilter
```

**All Implemented Interfaces:**: RevisionHandler

---

``` public class SERFileFilter extends javax.swing.filechooser.FileFilter implements RevisionHandler ```

SERFileFilter.java

**Version:**
:   $Revision: 1.3 $

**Author:**
:   Zhanna Melnikova-Albrecht (melnikov@cip.ifi.lmu.de), Rainer Holzmann (holzmann@cip.ifi.lmu.de)

---

**Constructor Summary**

`SERFileFilter(java.lang.String extension,
java.lang.String description)`


| **Method Summary** | |
| --- | --- |
| `boolean` | `accept(java.io.File f)`             Whether the given file is accepted by this filter. |
| `java.lang.String` | `getDescription()`             The description of this filter. |
| `java.lang.String` | `getRevision()`             Returns the revision string. |

**Methods inherited from class java.lang.Object**

`equals, getClass, hashCode, notify, notifyAll, toString, wait, wait, wait`

**Constructor Detail**

### SERFileFilter

```
public SERFileFilter(java.lang.String extension,
                     java.lang.String description)
```


**Method Detail**

### accept

```
public boolean accept(java.io.File f)
```

:   Whether the given file is accepted by this filter.

    :   **Specified by:**: `accept` in class `javax.swing.filechooser.FileFilter`

---


### getDescription

```
public java.lang.String getDescription()
```

:   The description of this filter.

    :   **Specified by:**: `getDescription` in class `javax.swing.filechooser.FileFilter`
    :   **See Also:**: `FileView.getName(java.io.File)`

---


### getRevision

```
public java.lang.String getRevision()
```

:   Returns the revision string.

    :   **Specified by:**: `getRevision` in interface `RevisionHandler`
    :   **Returns:**: the revision


---


|  |  |  |
| --- | --- | --- |
| **Overview** \| **Package** \| **Class** \| **Tree** \| **Deprecated** \| **Index** \| **Help** \| **Weka's home** | |  |
| **PREV CLASS**   **NEXT CLASS** | **FRAMES**    **NO FRAMES**     **All Classes** |  |
| SUMMARY: NESTED \| FIELD \| CONSTR \| METHOD | DETAIL: FIELD \| CONSTR \| METHOD |  |


---
